# Supplementary figures and images for: Genome-Wide Analysis of the Almond AP2/ERF Superfamily and Its Functional Prediction during Dormancy in Response to Freezing Stress
Source: Biology (Basel). 2022 Oct 17;11(10):1520. doi: 10.3390/biology11101520 (PMC9598233; doi:10.3390/biology11101520)

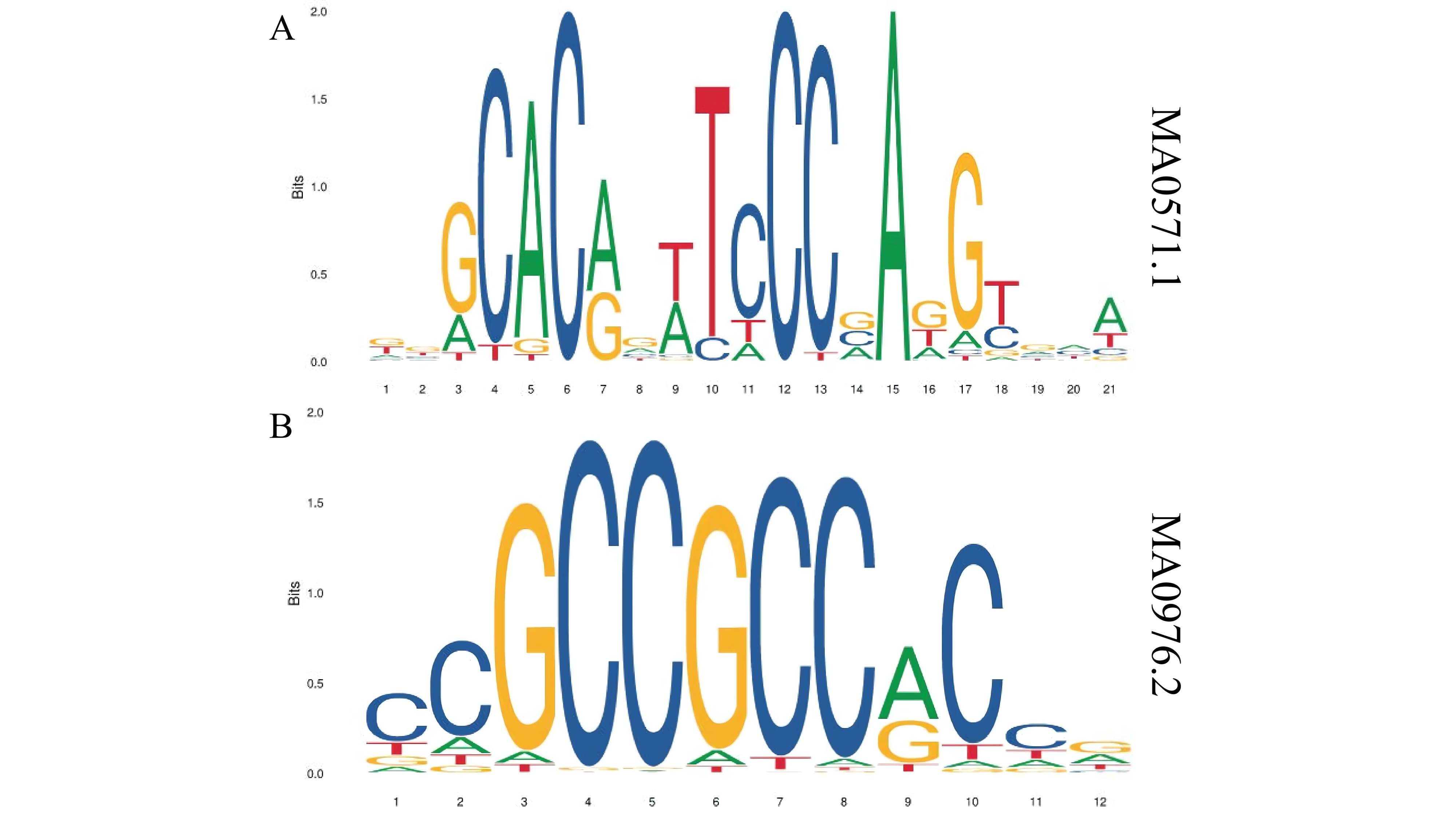

Supplement: Supplementary file 1 [file biology-11-01520-s001.zip › Figure S1 The consensus motif of the AP2 and ERFDREB DNA binding site from the JASPA CORE database.png]

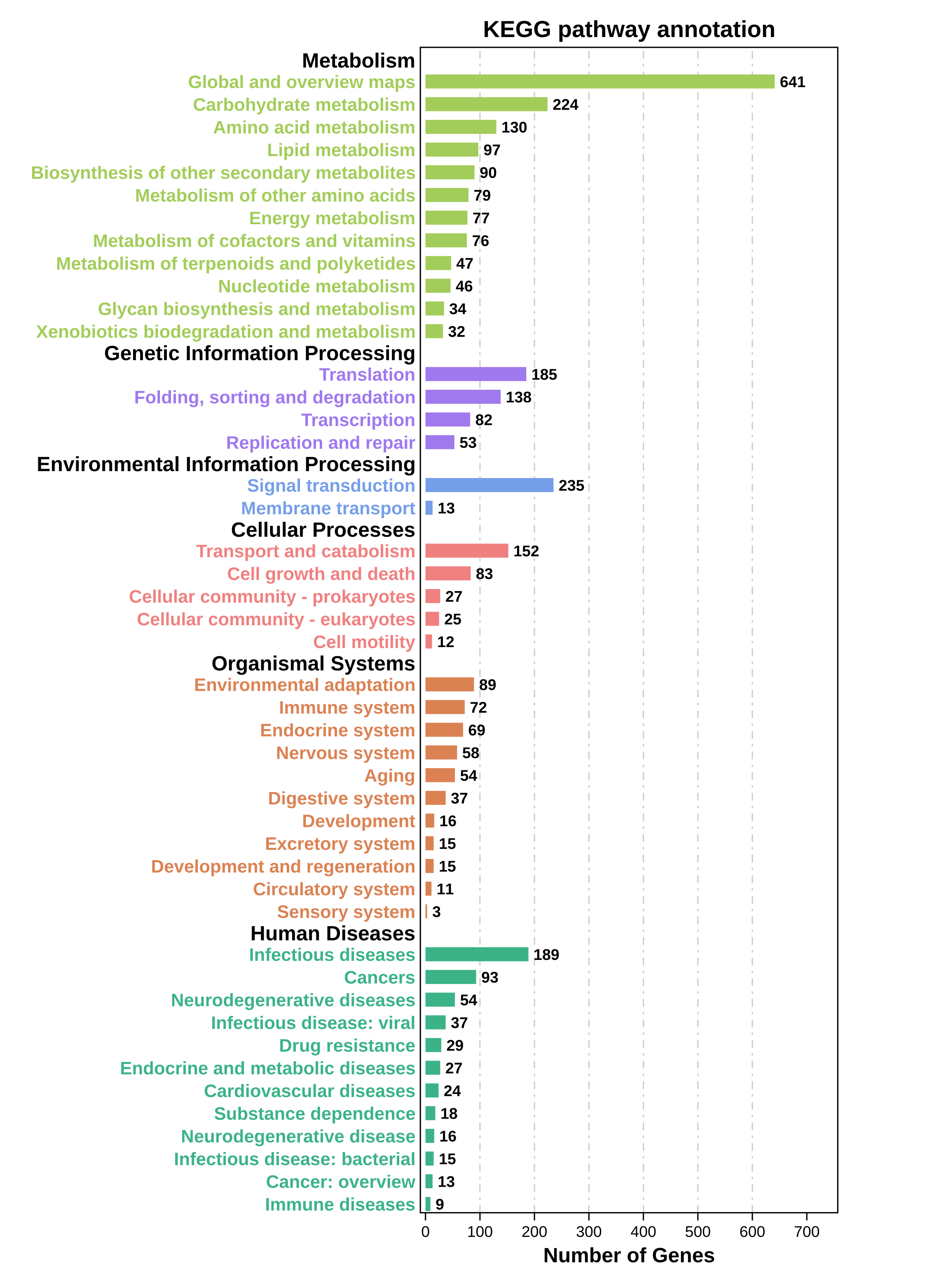

Supplement: Supplementary file 1 [file biology-11-01520-s001.zip › Figure S3 AP2 target genes protein KEGG enrichment results.png]

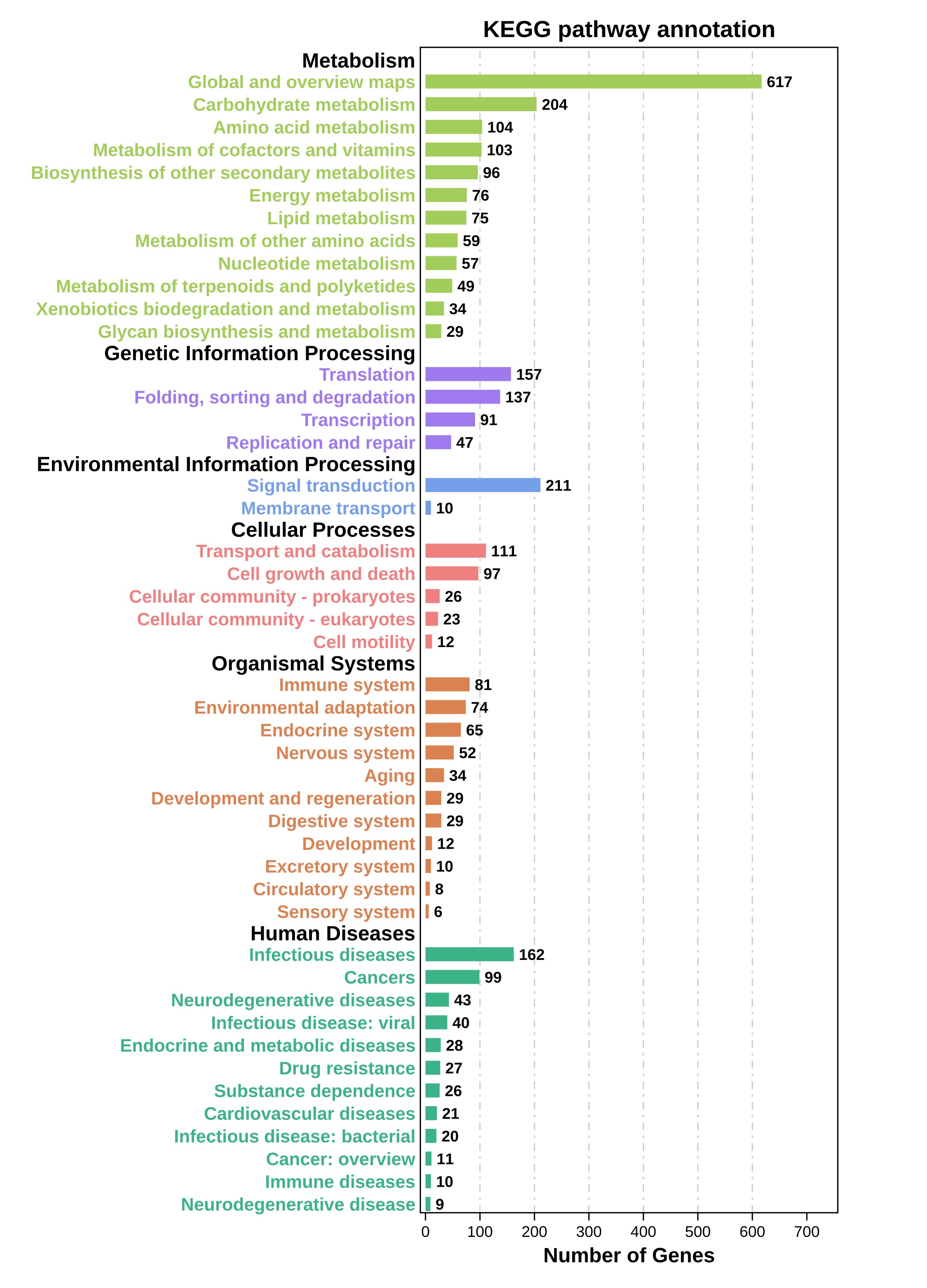

Supplement: Supplementary file 1 [file biology-11-01520-s001.zip › Figure S5 ERFDREB target genes protein KEGG enrichment results.png]
